# Development of a Ki-67-based clinical trial assay for neoadjuvant endocrine therapy response monitoring in breast cancer

**DOI:** 10.1007/s10549-017-4329-y

**Published:** 2017-06-13

**Authors:** Rodrigo Goncalves, Katherine DeSchryver, Cynthia Ma, Yu Tao, Jeremy Hoog, Maggie Cheang, Erika Crouch, Neha Dahiya, Souzan Sanati, Michael Barnes, Luis Otávio Zanatta Sarian, John Olson, Donald Craig Allred, Matthew J. Ellis

**Affiliations:** 1 Department of Obstetrics and Gynecology, State University of Campinas (UNICAMP), Campinas, Brazil; 2 Department of Medical Oncology, Breast Cancer Program, Washington University School of Medicine, St. Louis, MO USA; 3 Department of Biostatistics, Washington University School of Medicine, St. Louis, MO USA; 4 Clinical Trials and Statistic Unit, The Institute of Cancer Research, University of London, London, UK; 5 Department of Pathology and Immunology, Washington University School of Medicine, St. Louis, MO USA; 6 Ventana Medical Systems, Inc/Roche Diagnostics, Tucson, AZ USA; 7 Department of Surgery, University of Maryland, Baltimore, MD USA; 8 Lester and Sue Smith Breast Center, Baylor College of Medicine, One Baylor Plaza, Houston, TX 77030 USA

**Keywords:** Breast cancer, Biomarkers, Ki-67 proliferation marker

## Abstract

**Purpose:**

The recent publication of the ACOSOG Z1031 trial results demonstrated that Ki-67 proliferation marker-based neoadjuvant endocrine therapy response monitoring could be used for tailoring the use of adjuvant chemotherapy in ER+HER2-negative breast cancer patients. In this paper, we describe the development of the Ki-67 clinical trial assay used for this study.

**Methods:**

Ki-67 assay assessment focused on reproducing a 2.7% Ki-67 cut-point (CP) required for calculating the Preoperative Endocrine Prognostic Index and a 10% CP for poor endocrine therapy response identification within the first month of neoadjuvant endocrine treatment. Image analysis was assessed to increase the efficiency of the scoring process. Clinical outcome concordance for two independent Ki-67 scores was the primary performance metric.

**Results:**

Discordant scores led to a triage approach where cases with complex histological features that software algorithms could not resolve were flagged for visual point counting (17%). The final Ki-67 scoring approach was run on T1/2 N0 cases from the P024 and POL trials (*N* = 58). The percent positive agreement for the 2.7% CP was 87.5% (95% CI 61.7–98.5%); percent negative agreement 88.9% (95% CI: 65.3–98.6%). Minor discordance did not affect the ability to predict similar relapse-free outcomes (Log-Rank *P* = 0.044 and *P* = 0.055). The data for the 10% early triage CP in the POL trial were similar (*N* = 66), the percentage positive agreement was 100%, and percent negative agreement 93.55% (95% CI: 78.58–99.21%). The independent survival predictions were concordant (Log-rank *P* = 0.0001 and *P* = 0.01).

**Conclusions:**

We have developed an efficient and reproducible Ki-67 scoring system that was approved by the Clinical Trials Evaluation Program for NCI-supported neoadjuvant endocrine therapy trials. Using the methodology described here, investigators are able to identify a subgroup of patients with ER+HER2-negative breast cancer that can be safely managed without the need of adjuvant chemotherapy.

**Electronic supplementary material:**

The online version of this article (doi:10.1007/s10549-017-4329-y) contains supplementary material, which is available to authorized users.

## Introduction

Biomarkers of cell proliferation are used to assess prognosis and response to cancer treatment, and most clinical assays are based on Ki-67 immunohistochemistry (IHC) [1]. The Ki-67 nuclear protein is present in proliferating cells but absent in cells in G0 [2]. For breast cancer, Ki-67 analysis is relevant for estrogen receptor positive (ER+) early stage breast cancer [3–5] which presents as a spectrum of tumors with clinically indolent (Luminal A) or more aggressive features (Luminal B) [6]. While the “luminal” classification is based on gene expression analysis, a Ki-67 cut-point of 14% of cells staining positive has been proposed as a surrogate for the distinction between luminal A and luminal B [7]. This cut-point was considered clinically useful by the St. Gallen breast cancer consensus panel [8] but the concerns of the American Society of Clinical Oncology Tumor Marker Guideline Committee regarding the lack of rigor in Ki-67 scoring algorithms and the questionable validity of decision-making cut-points has slowed clinical implementation [9].

Ki-67 analysis also has potential for monitoring endocrine therapy response, which requires testing a tumor specimen after endocrine treatment has been initiated, for example, in surgical specimens after neoadjuvant aromatase inhibition [10]. The independent prognostic value of on-treatment Ki-67 was combined with pathologic tumor stage and ER status to develop the preoperative endocrine prognostic index (PEPI). A PEPI score of 0 (pT1/2N0, Ki-67 ≤2.7% and persistently expressed ER) was associated with such favorable long-term outcome after neoadjuvant endocrine therapy in the P024 trial [11] and IMPACT trial [10, 12] that chemotherapy was proposed to be unnecessary [13].

Recently, Ellis et al. published long-term follow-up results of the ACOSOG Z1031 trial in which clinical decisions were based on the PEPI score [14]. In ACOSOG Z1031 Cohort B, the authors tested the hypothesis that Ki-67-based algorithms can also address the concern that patients who are poorly responsive to neoadjuvant endocrine therapy should ideally be identified early for triage to alternate treatment, such as neoadjuvant chemotherapy or immediate surgery. The authors also successfully identified a subgroup of patients, based on PEPI scores that could be safely spared from adjuvant chemotherapy. In this paper, we describe the validation of Ki-67 cut-points relevant to neoadjuvant endocrine treatment monitoring and the development and validation of the Ki-67 clinical trial assay for prospective studies, used in ACOSOG Z1031 trial [14].

## Methods

### Database analysis for early Ki-67 cut-point for early triage to alternate treatment

Published data on research use only (RUO) quantitative polymerase chain reaction (qPCR)-based assignments of PAM50 luminal subtype (A vs. B) and RUO Ki-67 data from TMA analysis was made available from six hundred sixty-seven tumors with clinical ER-positive status from University of British Columbia. Of these tumors, 358 were classified as Luminal A and 309 as Luminal B [7]. Published Ki-67 data and clinical outcomes from the IMPACT trial [12] and POL Trial [15, 16] were used for the development of cut-points for prospective validation.

### Tumor samples for Ki-67 clinical trial assay development

For training the scanner and image analysis-based Ki-67 quantification algorithm, 61 node-positive samples from the P024 trial were examined. For assay validation for the early triage cut-point, core needle biopsies taken after 4 weeks of neoadjuvant endocrine therapy from 66 patients were accessed [15]. For validation of the 2.7% cut-point required for the PEPI score, surgical specimens from 58 patients with pathological stage 1 or 2A tumors were available from a combination of the POL trial [15] and the P024 trial [11].

### Ki-67 assay methodology

The research use only (RUO) Ki-67 assay employed to stain the P024 and POL samples for combined survival analysis employed the SP6 monoclonal antibody (Neomarkers) on a Shandon Sequenza^®^ Immunostainer using published methodology [13]. For the CLIA clinical trial assay, 5 micron sections from POL and P024 trials were subjected to H&E and Ki-67 staining in the CLIA-certified Washington University AMP laboratory using the CONFIRM anti-Ki-67 (30-9) rabbit monoclonal primary antibody as a pre-diluted reagent on a Benchmark XT platform according to the manufacturer instructions (Ventana, Tucson, AZ). Tonsil was used as the assay control.

### Ki-67 scoring approaches

For visual point counting (VPC), photomicrographs of three randomly selected fields were taken at 40X with a background grid and color printed (more fields to achieve the minimal cell count). Each observer counted both the total number tumor cells and the number of Ki-67-positive cells that intersect with first grid line. This process is repeated on every third gridline. All the cells on the slide were counted if three fields could not be obtained however at least 200 total tumor cells were required. For Ki-67 image analysis of the CLIA clinical trial assay, slides were scanned with the iScan Coreo scanner (Ventana). The computer image was reviewed and “Areas of Interest” (AOI) were selected at 4X magnification using the following guidelines: (1) identify the largest AOI of representative clear invasive tumor; (2) exclude DCIS, vessels, lymphocytes; (3) avoid AOIs in peri-necrotic or necrotic areas; (4) identify at least 3 AOIs and a maximum of 10. The image analysis was performed using the FDA cleared VENTANA Companion Algorithm Ki-67 (30-9) and the VENTANA VIRTUOSO software (Roche).

### Assessment of concordance

Two pathologists, blinded to each other’s data and any data from earlier analyses of the samples, independently reviewed the Ki-67 slide scans and identified AOI for either image analysis or VPC methodology. Similarly blinded trained technicians generated the VPC Ki-67 percentage.

### Statistical analysis

Analysis of variance using a scatter-plot analysis was calculated using Pearsons’ correlation and Spearman correlation coefficients. Two pathologist concordance for the 2.7 and 10% cut-points were analyzed using four-by-four contingency table analysis, simple Kappa coefficients and percent positive and negative agreements. The prognostic effect of modified PEPI 0 (pT1/2, N0, Ki-67 ≤ 2.7%) vs. non-0 assignments based on the CLIA Ki-67 assay determined using the Kaplan–Meier method. The log-rank test was conducted to examine statistical significance. Similar analyses were performed to correlate survival outcomes of patients with early on-treatment Ki-67 (>10 vs ≤ 10%) in the POL trial. Bland–Altman plots were generated to assess bias between pathologists.

## Results

### A Ki-67-based definition of poorly endocrine therapy responsive tumors for triage to alternate treatment

**Fig. 1:**
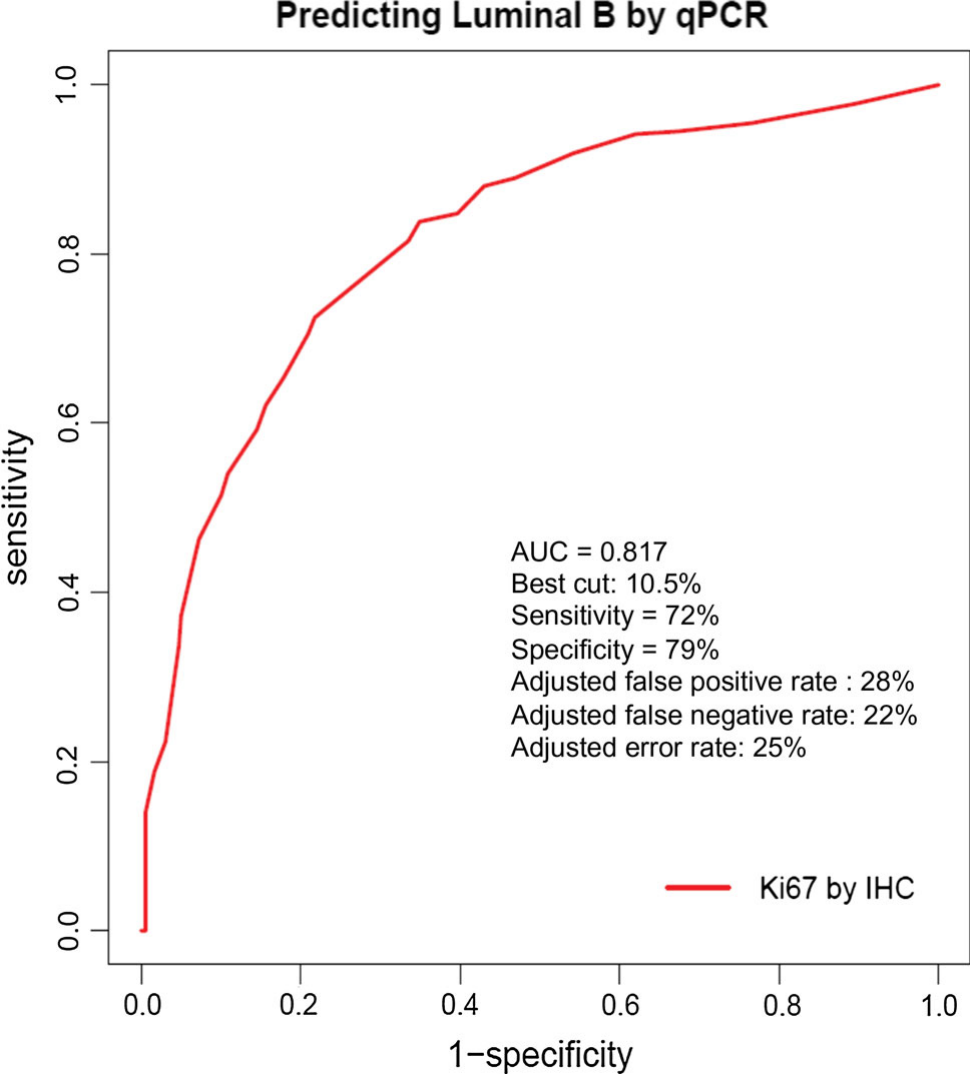
ROC curve to determine the best Ki-67 cut-point to differentiate Luminal A breast cancer from Luminal B breast cancer based on a PAM50 qPCR RUO assay

To develop a Ki-67-based approach for the early identification of non-responders within a month of starting treatment, we examined the interaction between baseline Ki-67 levels and a qPCR-PAM50-based definition of luminal A versus luminal B breast cancer using published data [17]. Using ROC methodology, a 10% Ki-67 cut-point of Ki-67 best served as a surrogate for the genomic luminal definitions in this data set (Fig. 1). We therefore hypothesized that tumors with an early Ki-67 value above 10% despite endocrine therapy would be enriched for endocrine therapy resistant, luminal B-type tumors with a high relapse rate. This is supported by the early on-treatment data from the POL [15] and IMPACT [12] trials which indicated that Ki-67 levels >10% predicted a higher level of Ki-67 in the surgical sample, a higher PEPI score, a smaller number of patients in the PEPI-0 group, and worse RFS [14].

### PEPI score validation and modification

**Fig. 2:**
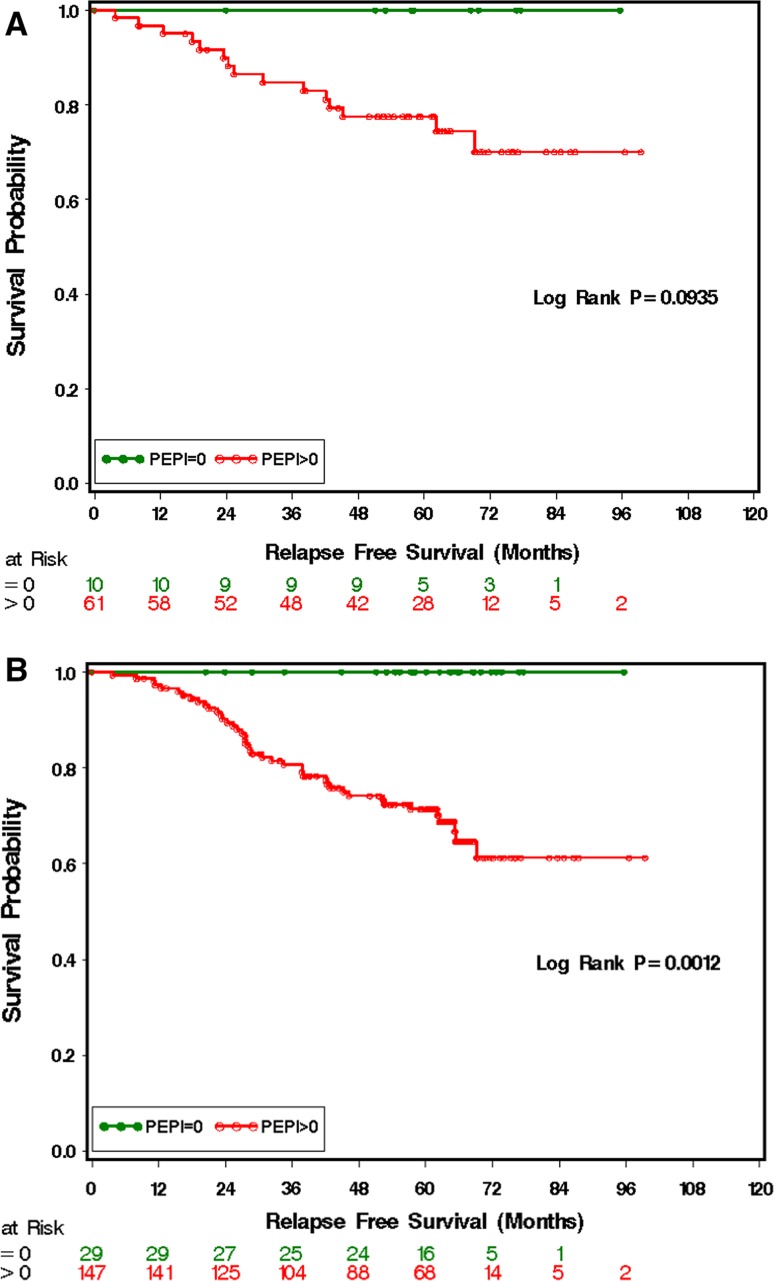
Kaplan–Meier curves showing relapse-free survival in the POL trial (**a**) and in the combined data from the POL/P024 trials using an RUO assay and VPC scoring methodology (**b**). *PEPI* Preoperative endocrine prognostic index

Long-term outcomes from the POL trial provided an opportunity to further validate of the PEPI score. While the number of cases was modest, no relapses were observed in 10 patients with PEPI 0 tumors after a median follow-up of 59 months (Fig. 2a). We also developed a modified PEPI score that did not include ER status at surgery, because of clinical trial proposals that included the use of the estrogen receptor down-regulator fulvestrant, the use of which confounds the interpretation of ER levels after treatment initiation [18]. In the P024, IMPACT and POL trials, patients with modified PEPI score of 0 were all ER+ (Allred score 3–8) because ER Allred score 0–2 post aromatase inhibitor or tamoxifen treatment was associated with either a high Ki-67 or high tumor staging (or both) excluding these cases from PEPI-0 status without the need for information on ER. In the combined P024 trial/POL trial data, no relapses were observed in the 29 patients (19 pT1N0, 10 pT2N0) with modified PEPI-0 status (i.e., without scoring ER) during a median follow-up of 62.5 months (Fig. 2b).

### Validation of visual point counting (VPC) for outcome prediction after neoadjuvant endocrine therapy

**Fig. 3:**
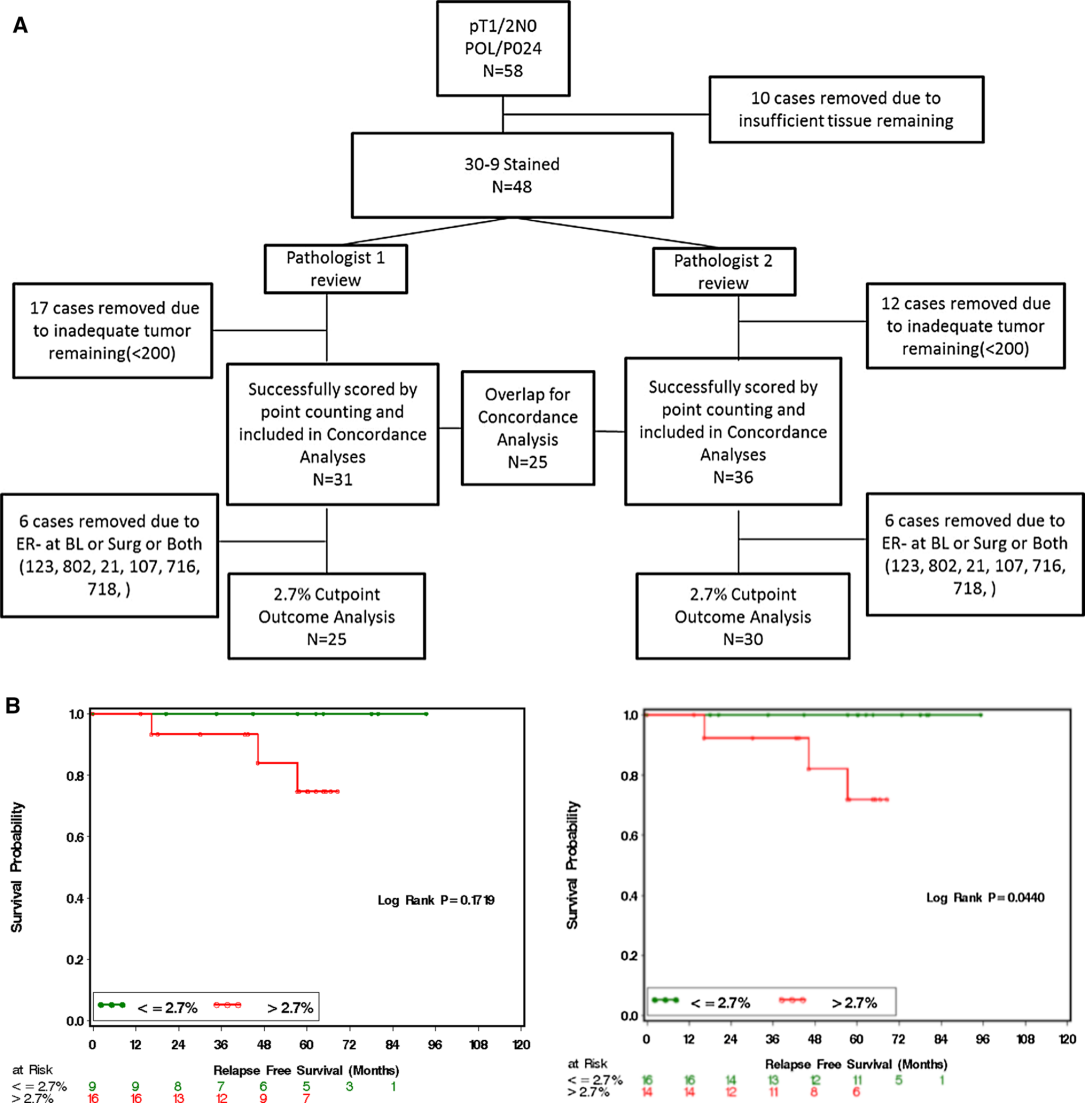
**a** REMARK diagram showing sample flow through the study for validation of the visual point counting technique. **b** Kaplan–Meier curves from two independent pathologists demonstrating relapse-free survival according to Ki-67 score >2.7 or ≤2.7%

In previous analyses, VPC methodology was routinely used but this approach had not been formally assessed as part of a clinical trial assay. Available surgical tumor samples from pT1/2 N0 cases in the POL and P024 trials were therefore stained using the commercial 30-9 antibody assay in a CLIA-certified laboratory. Stage 1 or 2A cases were chosen because a Ki-67 cut-point (CP) of ≤2.7% is the only factor that determines the modified PEPI score of 0. The REMARK sample flow chart for the duplicate study is provided in Fig. 3a. Outcome predictions were reproducible, with no relapses observed for patients assigned modified PEPI 0 (Ki-67 ≤ 2.7%) status by either pathologist (Fig. 3b). Analysis of Ki-67 as a continuous variable indicated that the Spearman Correlation Coefficient was 0.938 (*p* < 0.0001) (Figure S2A), and there was no trend for increased discordance across the range of Ki-67 values (Figure S2B). The positive CP agreement was 13/13 (100%). The negative agreement was 9/12 (0.75) (95% exact confidence limit: 0.428–0.945). Simple Kappa Coefficient was 0.7573 (95% Confidence limit: 0.5073; 1) (Table 1, S1A).

**Table 1:** Summary of Ki-67 scoring agreement statistics according to the sample sets and different counting methods used

Sample set	Ki-67 Cut-point (%)	Counting method	Percent positive agreement (95% CI)	Percent negative agreement (95% CI)	Kappa coefficient (95% CI)
Validation set	2.7	Visual point counting	100	75 (42.8;94.5)	0.76 (0.51–1)
Training set	2.7	Virtuoso software	96.7 (82.8–99.9)	88.5 (69.9; 97.6)	0.85 (0.72;0.99)
	10	Virtuoso software	100	97.9 (88.7;1)	0.94 (0.81;1)
Validation set	2.7	Ki-67 SOP	87.5 (61.7;98.5)	88.9 (65.3;98.6)	0.76 (0.55;0.98)
	10	KI-67 SOP	100	93.6 (78.6;99.2)	0.86 (0.66;1)

### Assessment of an image analysis approach for Ki-67 scoring

**Fig. 4:**
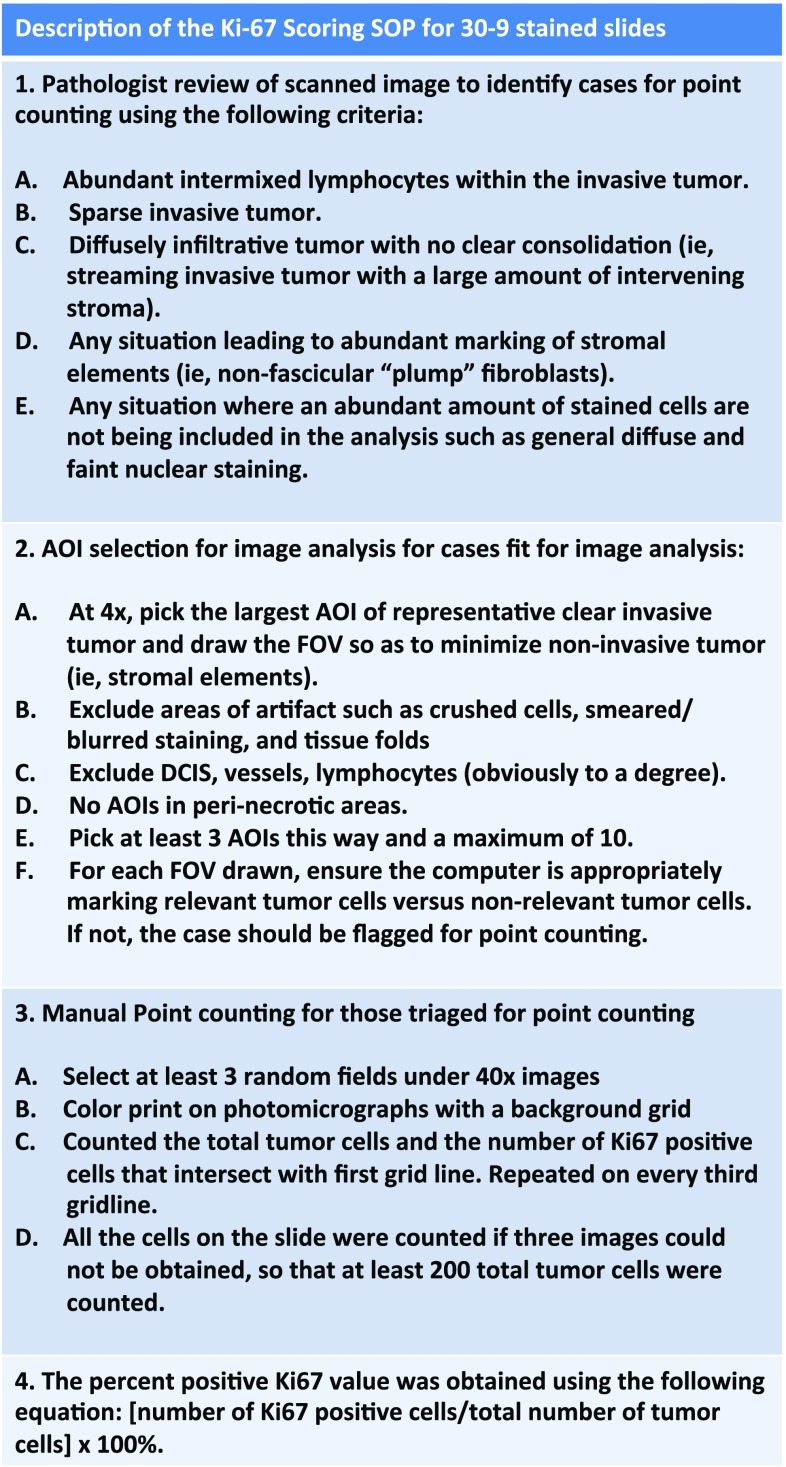
Standard operating procedure (SOP) for Ki-67 scoring with the aid of an image scanner and the Companion Algorithm image analysis software

The performance of VPC, while technically adequate, is laborious and therefore not ideal for real-time clinical reporting. We therefore considered a Ki-67 scoring approach using an FDA cleared scanner and image interpretation software to determine if these tools were appropriate. For training, the 30-9 antibody-based commercial assay was conducted on 61 surgical samples from patients with node-positive disease in the P024 trial (Figure S1). The slides were scanned and then analyzed by two pathologists who independently reviewed the images and drew areas of interest (AOI) for Ki-67 scoring. In five instances, the algorithm did not accurately differentiate between benign and malignant cells. These cases were noteworthy for abundant lymphocyte infiltration, sparse tumor cells where tumor cells were streaming through the tissue with a large amount of intervening stroma, abundant marking of non-fascicular “plump” fibroblasts, or when the Ki-67 stain was generally diffuse and nuclear staining was faint. Excluding these cases, the Spearman Correlation Coefficient was 0.89 (*p* < 0.0001) (Figure S3A). The Bland–Altman plot showed no bias in scoring between the two pathologists across the range of Ki-67 values (Figure S3B). The CP concordance was then analyzed. For the 2.7% cut-point, the positive agreement was 29/30 (0.96) (95% exact confidence limit: 0.82–0.99). The negative agreement was 23/26 (0.88) (95% exact confidence limit: 0.69–0.97). The kappa coefficient was 0.85 (95% confidence limit: 0.71; 0.99). Using the 10% cut-point, the positive agreement was 100%, and the negative agreement was 46/47 (0.97) (95% exact confidence limit: 0.88–1). The kappa coefficient was 0.93 (95% Confidence limit: 0.81; 1.0) (Table 1, S1B). A “locked-down” scoring standard operating procedure (SOP) was generated that included an option to triage to VPC if the pathology was judged too complex for the scanner to differentiate benign from malignant cells (Fig. 4).

### Validation of combined imaging/VPC Ki-67 scoring SOP for the 2.7% Ki-67 cut-point

**Fig. 5:**
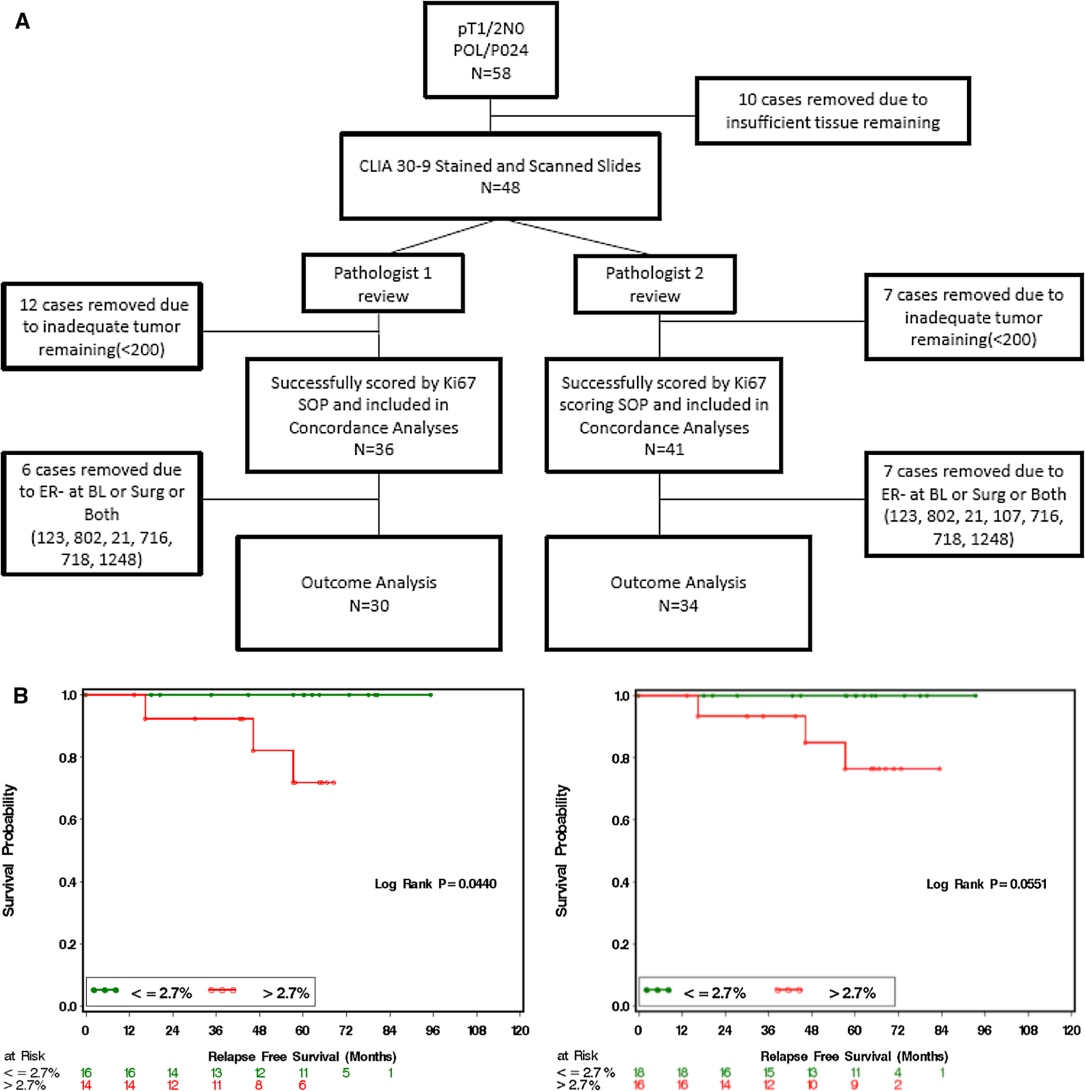
**a** REMARK diagram showing patient flow through the study for validation of the standard operating procedure for Ki-67 scoring. **b** Kaplan–Meier curves from two independent pathologists demonstrating relapse-free survival according to Ki-67 score ≤2.7 or >2.7%

To validate the combined imaging/VPC SOP for Ki-67 scoring, the CLIA assay stained slides used for the VPC assessment were scanned and independently assessed by two pathologists. The sample flow chart is shown in Fig. 5a. Kaplan–Meier analysis by modified PEPI 0 is shown in Fig. 5b for the two separate scoring exercises. Again, no relapses were observed in patients with modified PEPI 0 during the follow-up using this scoring method from either pathologist. Continuous data analysis indicated that the Spearman Correlation Coefficient was 0.86 (*p* < 0.0001) (Figure S4A). No scoring bias was observed across the scored range (Figure S4B). The percentage positive agreement between the two pathologists in scoring the 2.7% CP Ki-67 using the SOP was 0.87 (95% CI 0.61–0.98). The negative agreement was 0.88 (95% CI 0.65–0.98). Simple kappa coefficient was 0.76 (95% CI 0.54–0.98) (Table 1, S1C).

### Validation of combined imaging/VPC Ki-67 scoring SOP for the 10% Ki-67 cut-point

**Fig. 6:**
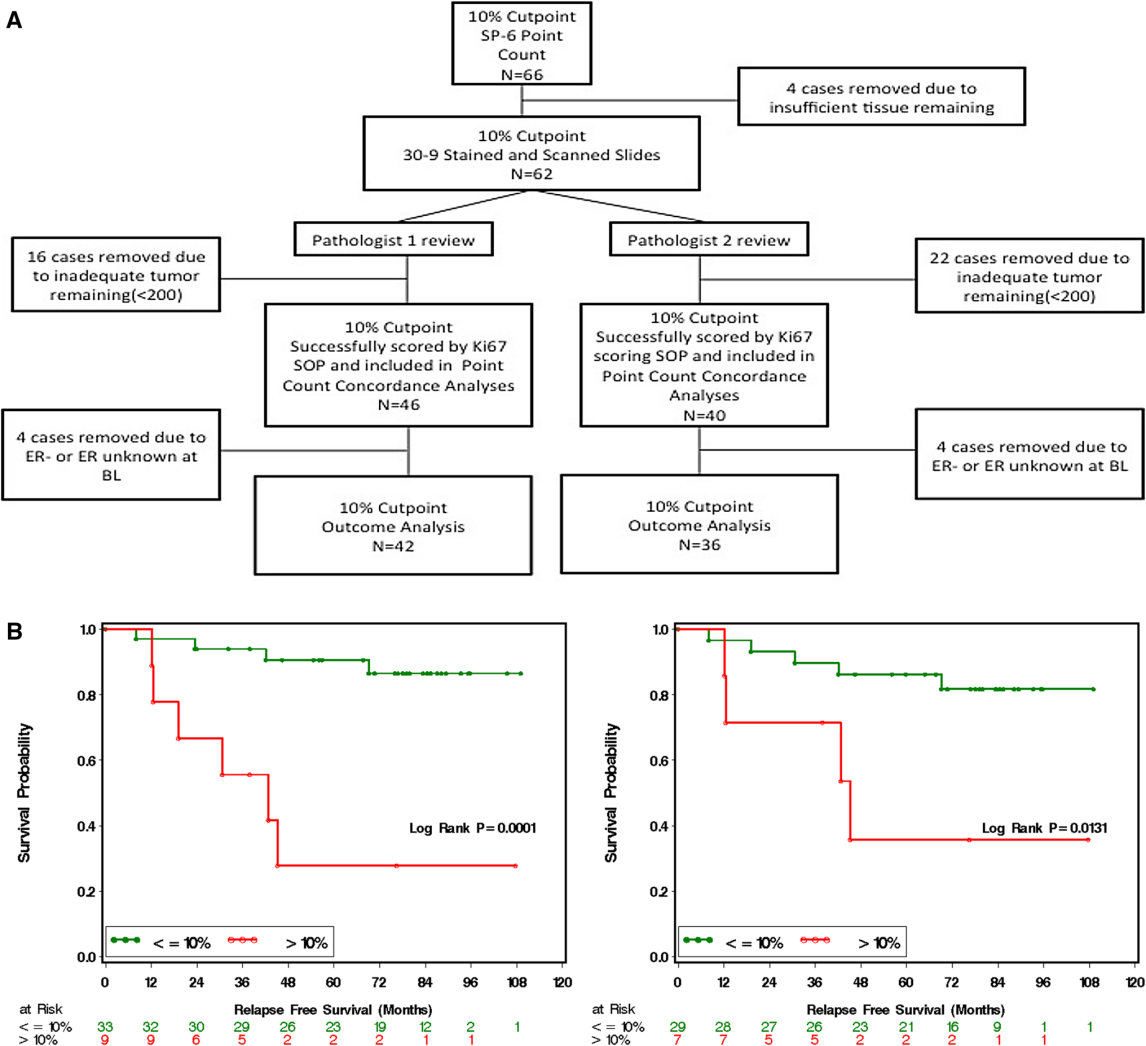
**a** REMARK diagram showing patient flow through the study for validation of the standard operating procedure for Ki-67 scoring. **b** Kaplan–Meier curves from two independent pathologists demonstrating relapse-free survival according to Ki-67 score ≤10 or >10% (**b**)

To validate the combined imaging/VPC approach for the 10% cut-point one-month biopsies from the POL trial were stained using the Ki-67 30-9 clinical trial assay, scanned, and then independently reviewed for algorithm accuracy and independently scored by two pathologists. The REMARK sample flow chart is shown in Fig. 6a. Concordant Kaplan–Meier analyses for the 10% cut-point for two separate scoring exercises are shown in Fig. 6b. The poor outcome for patients in the >10% category was reproducible. The Spearman Correlation Coefficient was 0.86 (*p* < 0.0001) (Figure S5A). No scoring bias was observed across the scoring range (Figure S5B). The percentage positive agreement between the two pathologists in scoring the 10% CP Ki-67 using the SOP was 100%. The negative agreement was 93.6 (78.6–99.2). The kappa coefficient was 0.86(0.66–1) (Table 1, S1D).

## Discussion

We have developed an efficient and reproducible Ki-67 scoring system that was approved by the Clinical Trials Evaluation Program (CTEP) for NCI-supported neoadjuvant endocrine therapy trials. The combination of image analysis with triage to VPC, when deemed necessary, respects the finding that the image analysis software does not always differentiate between certain types of normal and malignant cells. This approach also emphasizes the critical role of the pathologist in the review of the scanned images to determine the most appropriate scoring approach (image analysis or VPC) when the histology is complex. The sample flow charts illustrate that while pathologists may have different interpretations for the requirement for visual point counting, these differences do not strongly affect clinical outcome prediction. The VPC triage rate was, on average 17%, demonstrating that the image analysis approach can be used in the majority of cases, markedly reducing the need to conduct laborious VPC to a manageable number of cases. A weakness of our study is that the sample sets were denuded by earlier analyses and produced very modest sample sizes, and therefore, our analysis did not produce evidence for immediate clinical utility. However, the Ki-67 clinical trial assay developed and described in this paper was further validated in ACOSOG Z1031A study. In that trial, with a median follow-up of 5.5 years, this Ki67 methodology was able to identify a subgroup of patients with PEPI score = 0 (Ki-67 ≤ 2.7%, T1/2, N0) that were safely managed without adjuvant chemotherapy. Among patients with PEPI = 0 score that were managed without chemotherapy, only 4 out of 119 presented with a relapse during follow-up. The triage rate to VPC in the Z1031A trial was 6%, even lower than what we found in the POL and P024 sample sets.

An issue not addressed in the scoring algorithm proposed herein concerns cases where the Ki-67 staining is not uniform—our VPC or image analysis approach requires random fields. We consider a Ki-67 heterogeneity-agnostic approach equivalent to genomic approaches that also do not clearly respect tissue heterogeneity. While analysis of heterogeneity, or “Ki-67 hot spot” analysis, should be pursued, this is a complex problem that will require the development of a “hot-spot” definition that can be shown to drive outcome more effectively than an analysis of all the tumor cells in the section.

Another point of controversy is the Ki-67 cut-point as a surrogate for luminal A versus luminal B breast cancer. In our current analysis, 10% has the best operating characteristics while an earlier publication on a different dataset using similar methodology suggested 14% [7], which suggests a narrow range of values for this purpose. From the perspective of this paper, the 10% cut-point was more conservative and serves the purpose of early identification of patients with luminal B-type tumors with endocrine therapy resistance characteristics well. The rapid onset of advanced disease for patients with Ki-67 > 10% despite aromatase inhibitor therapy (see Fig. 6b for example) underscores the importance of developing a robust clinical trial strategy for this high-risk population.

When we submitted our Ki-67 clinical trial assay to the FDA they ruled the proposed treatment algorithms as “no significant risk” because Ki-67 analysis actually reduces the risk of under-treatment. This conclusion was based on the analysis of chemotherapy use according to PEPI score shows that when medical oncologists rely on pathological stage alone after neoadjuvant endocrine therapy most patients with low stage do not receive chemotherapy. Combined analysis of the P024, IMPACT and POL trials showed that only 8% of patients with pathological stage 1 or 2A disease received adjuvant chemotherapy (Table S2). Thus, the FDA considered that knowledge of the Ki-67 value in the pathological specimen reduced the risk of under-treatment for patients with low pathological stage tumors but aggressive biological characteristics (high on-treatment Ki-67).

Even though ASCO still does not support Ki67 in its clinical guidelines, a recent editorial acknowledges our team’s efforts as “an important step in the direction of clinical respectability for Ki67 as a useful breast cancer prognosticator” [19]. The next necessary step is already being taken as the Ki-67 clinical trial assay we described in this paper is being prospectively validated in the ALTERNATE trial (NCT01953588).

## Electronic supplementary material

Below is the link to the electronic supplementary material.

Supplementary material 1 (DOCX 35 kb)

Supplementary material 2 (DOCX 23 kb)

Supplementary material 3 (PDF 34 kb)

Supplementary material 4 (PDF 75 kb)

Supplementary material 5 (PDF 94 kb)

Supplementary material 6 (PDF 93 kb)

Supplementary material 7 (PDF 95 kb)
