# Supplementary material for: Development of a Ki-67-based clinical trial assay for neoadjuvant endocrine therapy response monitoring in breast cancer
Source: Breast Cancer Res Treat. 2017 Jun 13;165(2):355–64. doi: 10.1007/s10549-017-4329-y (PMC5543203; doi:10.1007/s10549-017-4329-y)
Supplement: Supplementary file 1 — Supplementary material 1 (DOCX 35 kb) [file 10549_2017_4329_MOESM1_ESM.docx]

A)

| Two Pathologist Concordance in Ki67 visual point counting of CLIA 30-9 stained Breast Cancer surgical samples from pT1/2N0 cohort of P024/POL trial | | | |
| --- | --- | --- | --- |
| 2.7% cut point | Pathologist 2 | | |
| Pathologist 1 | > 2.7% | < 2.7% | Total |
| > 2.7% | 13 | 3 | 16 |
| < 2.7% | 0 | 9 | 9 |
| Total | 13 | 12 | 25 |

B)

| Two Pathologist Concordance in Ki67 image analysis of CLIA 30-9 stained Breast Cancer surgical samples from the Node Positive Cohort of P024 trial. | | | |
| --- | --- | --- | --- |
| 2.7% cut point | Pathologist 2 | | |
| Pathologist 1 | >2.7% | < 2.7% | Total |
| >2.7% | 29 | 3 | 32 |
| < 2.7% | 1 | 23 | 24 |
| Total | 30 | 26 | 56 |
|  | | | |
| 10% cut point | Pathologist 2 | | |
| Pathologist 1 | > 10% | < 10% | Total |
| >10% | 9 | 1 | 10 |
| < 10% | 0 | 46 | 46 |
| Total | 9 | 47 | 56 |

C)

| Two Pathologist Concordance for the final Ki67 SOP scoring of the CLIA 30-9 stained pT1/2N0 cohort of P024/POL trial | | | |
| --- | --- | --- | --- |
| 2.7% cut point | Pathologist 2 | | |
| Pathologist 1 | > 2.7% | < 2.7% | Total |
| > 2.7% | 14 | 2 | 16 |
| < 2.7% | 2 | 16 | 18 |
| Total | 16 | 18 | 34 |

D)

| Two Pathologist Concordance for the Ki67 SOP scoring of the CLIA 30-9 stained 4-week biopsy in POL trial | | | |
| --- | --- | --- | --- |
| 10% cut point | Pathologist 2 | | |
| Pathologist 1 | > 10% | < 10% | Total |
| > 10% | 8 | 2 | 10 |
| < 10% | 0 | 29 | 29 |
| Total | 8 | 31 | 39 |
