# Supplementary material for: Development of a Ki-67-based clinical trial assay for neoadjuvant endocrine therapy response monitoring in breast cancer
Source: Breast Cancer Res Treat. 2017 Jun 13;165(2):355–64. doi: 10.1007/s10549-017-4329-y (PMC5543203; doi:10.1007/s10549-017-4329-y)
Supplement: Supplementary file 2 — Supplementary material 2 (DOCX 23 kb) [file 10549_2017_4329_MOESM2_ESM.docx]

| Chemotherapy administration in the P024, IMPACT and POL trials | | | | |
| --- | --- | --- | --- | --- |
| P024\PEPI | 0 | 1-3 | 4+ | Total |
| Chemotherapy | 5 (12%) | 24 (37%) | 28 (54%) | 57 |
| Total | 41 | 65 | 52 | 158 |
|  | | | | |
| IMPACT\PEPI | 0 | 1-3 | 4+ | Total |
| Chemotherapy | 1 (3%) | 21 (22%) | 26 (35%) | 48 |
| Total | 31 | 97 | 75 | 203 |
|  | | | | |
| POL\PEPI | 0 | 1-3 | 4+ | Total |
| Chemotherapy | 1 (9%) | 16 (57%) | 24 (67%) | 41 |
| Total | 11 | 28 | 36 | 75 |
