# Supplementary material for: Development of a Ki-67-based clinical trial assay for neoadjuvant endocrine therapy response monitoring in breast cancer
Source: Breast Cancer Res Treat. 2017 Jun 13;165(2):355–64. doi: 10.1007/s10549-017-4329-y (PMC5543203; doi:10.1007/s10549-017-4329-y)
Supplement: Supplementary file 3 — Supplementary material 3 (PDF 34 kb) [file 10549_2017_4329_MOESM3_ESM.pdf]

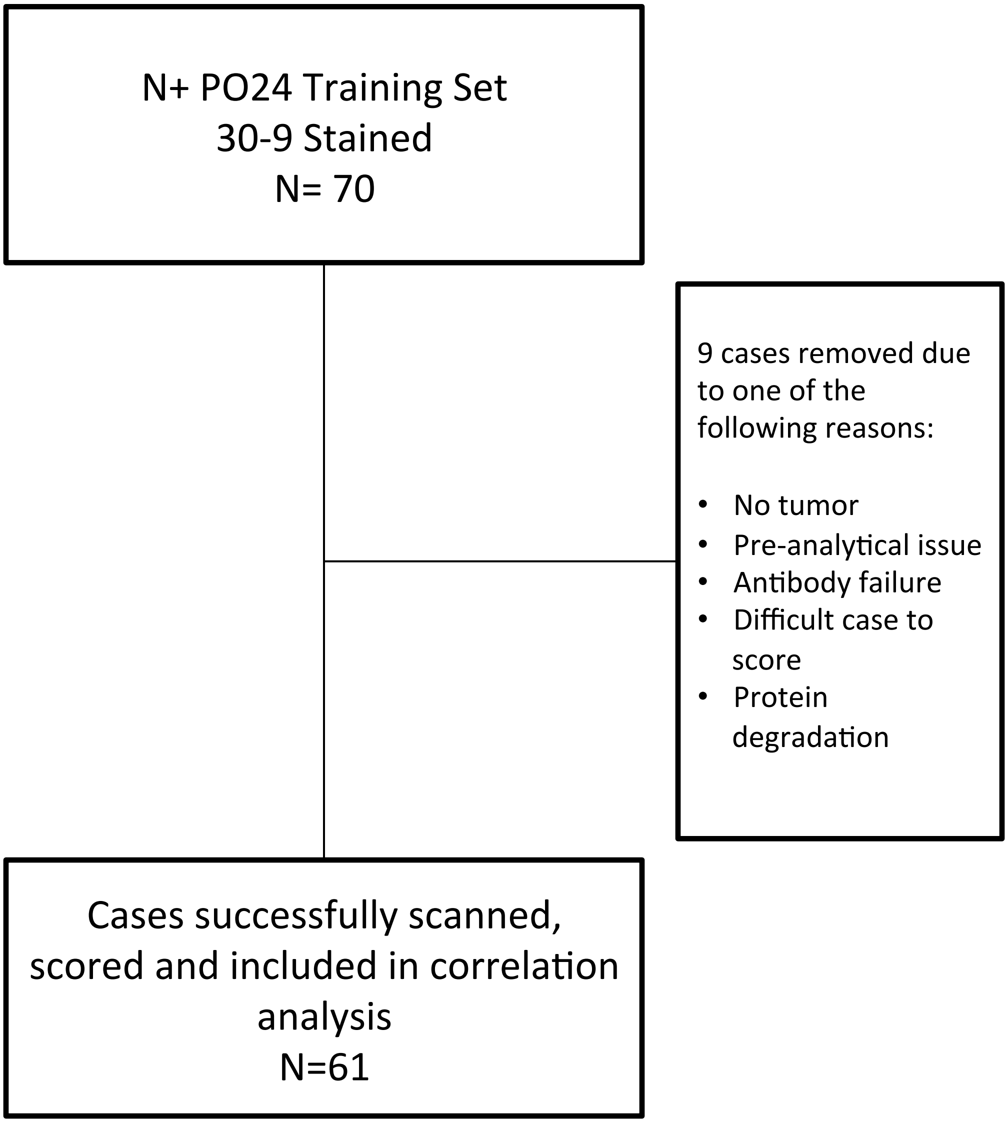
N+ PO24 Training Set
30-9 Stained
N= 70
9 cases removed due to one of the following reasons:
• No tumor
• Pre-analytical issue
• Antibody failure
• Difficult case to score
• Protein degradation
Cases successfully scanned, scored and included in correlation analysis
N=61
