# Supplementary figures and images for: Development of a Ki-67-based clinical trial assay for neoadjuvant endocrine therapy response monitoring in breast cancer
Source: Breast Cancer Res Treat. 2017 Jun 13;165(2):355–64. doi: 10.1007/s10549-017-4329-y (PMC5543203; doi:10.1007/s10549-017-4329-y)

A

Scatter plot

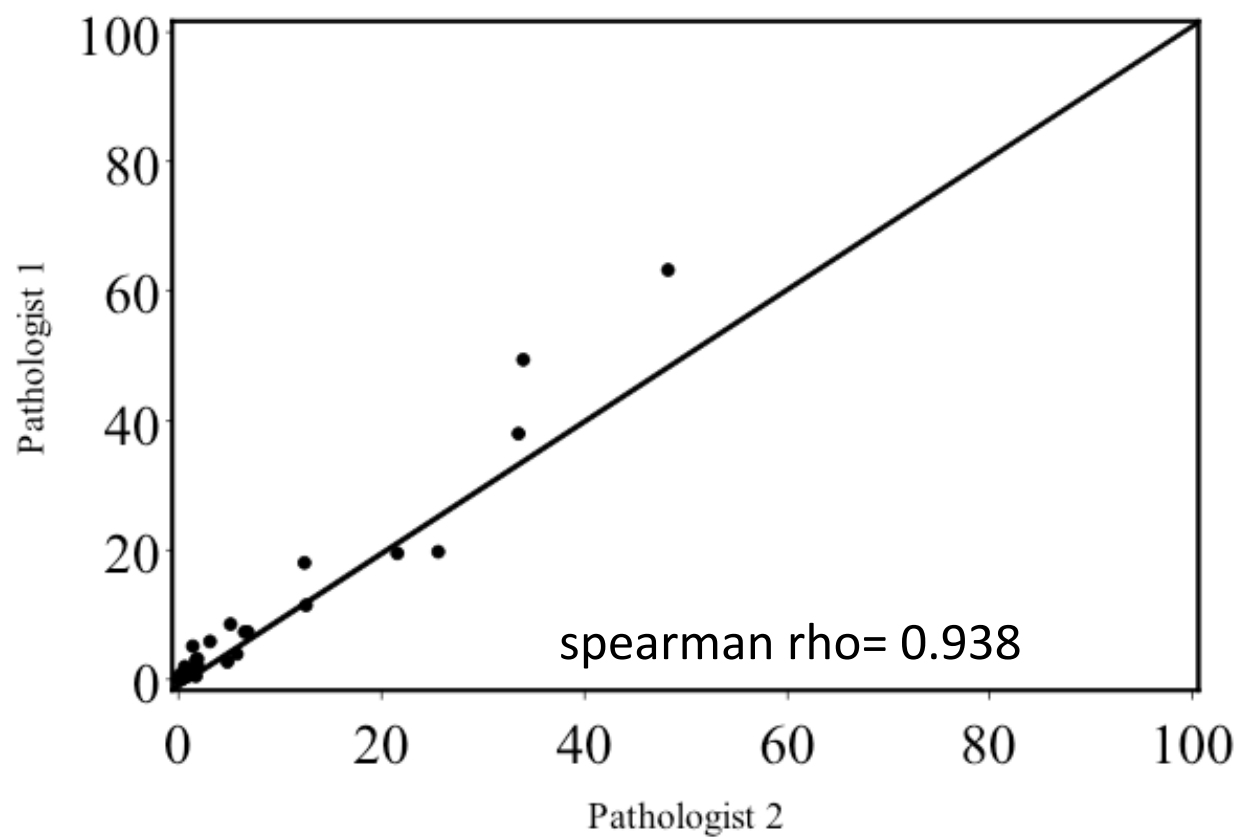

B

Bland Altman plot

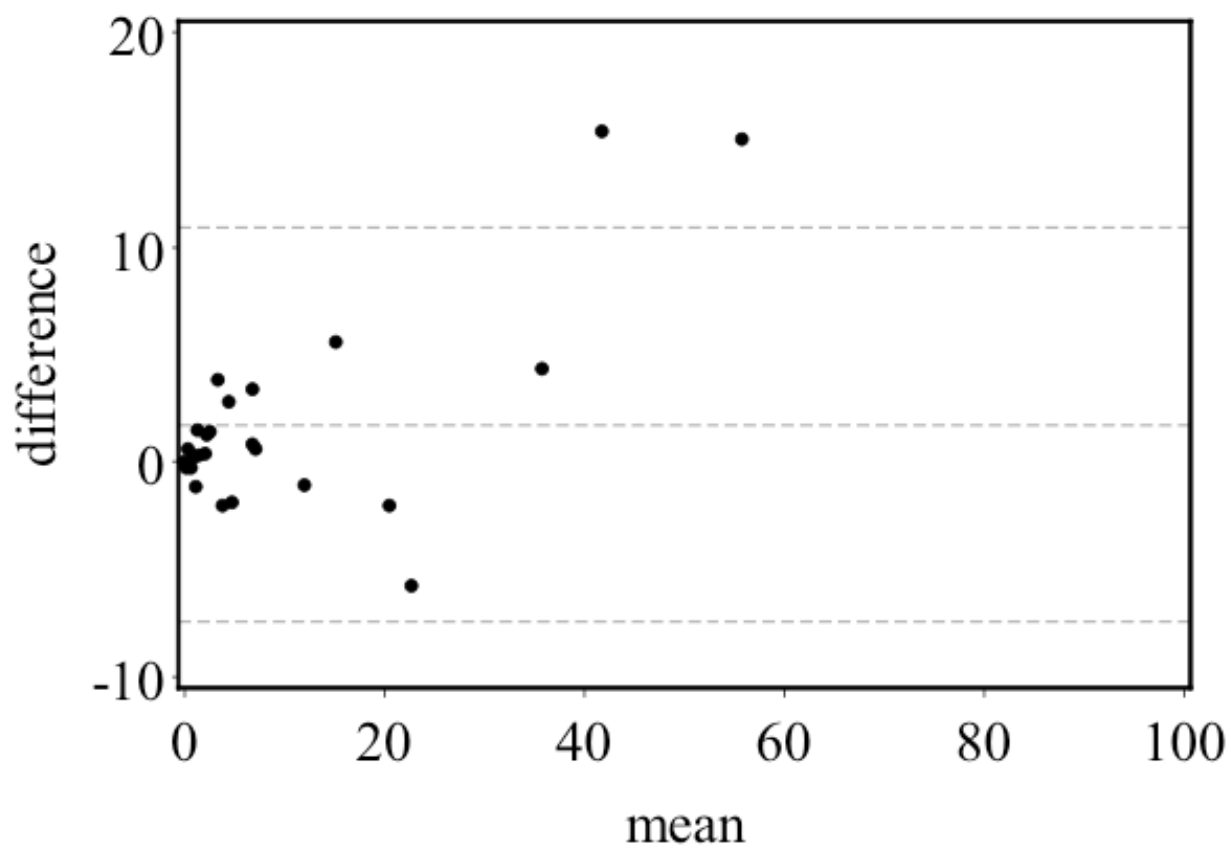

Supplement: Supplementary file 4 — Supplementary material 4 (PDF 75 kb) [file 10549_2017_4329_MOESM4_ESM.pdf]

A

Scatter plot

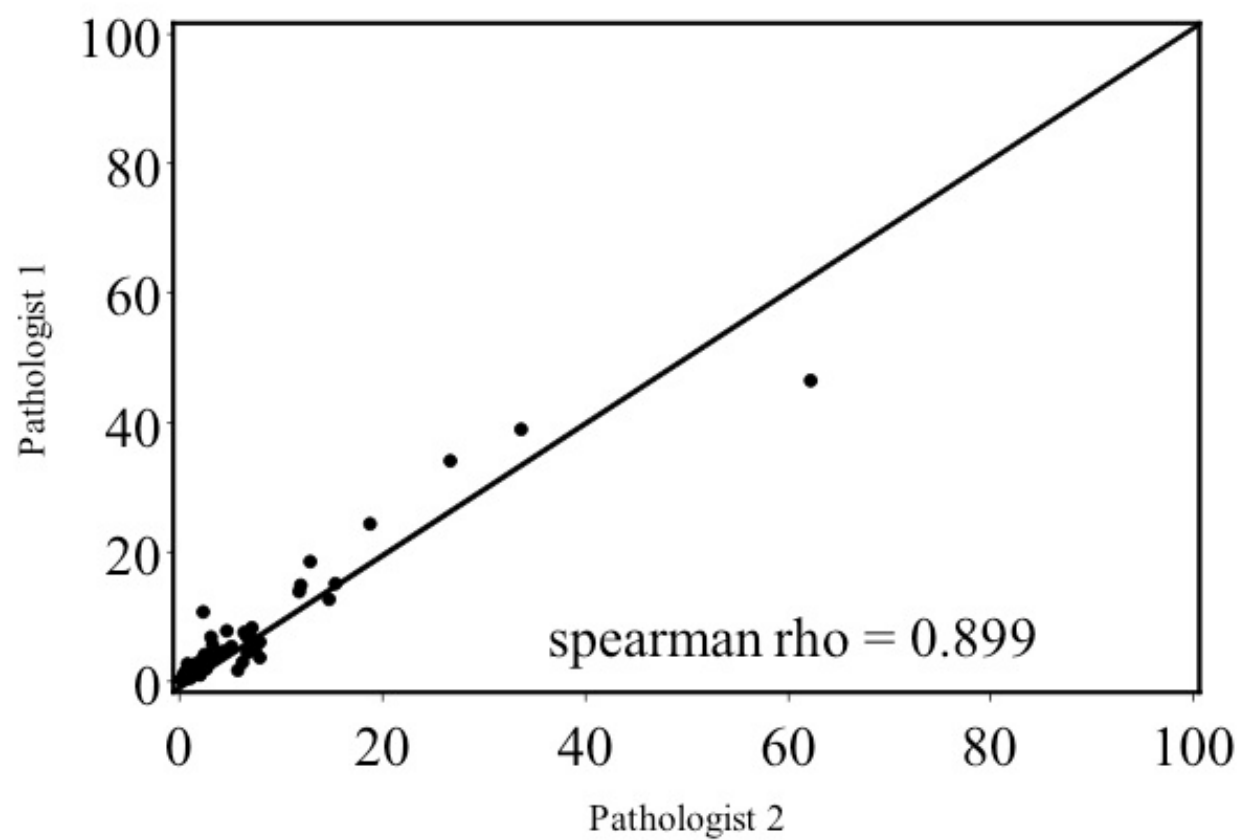

B

Bland Altman plot

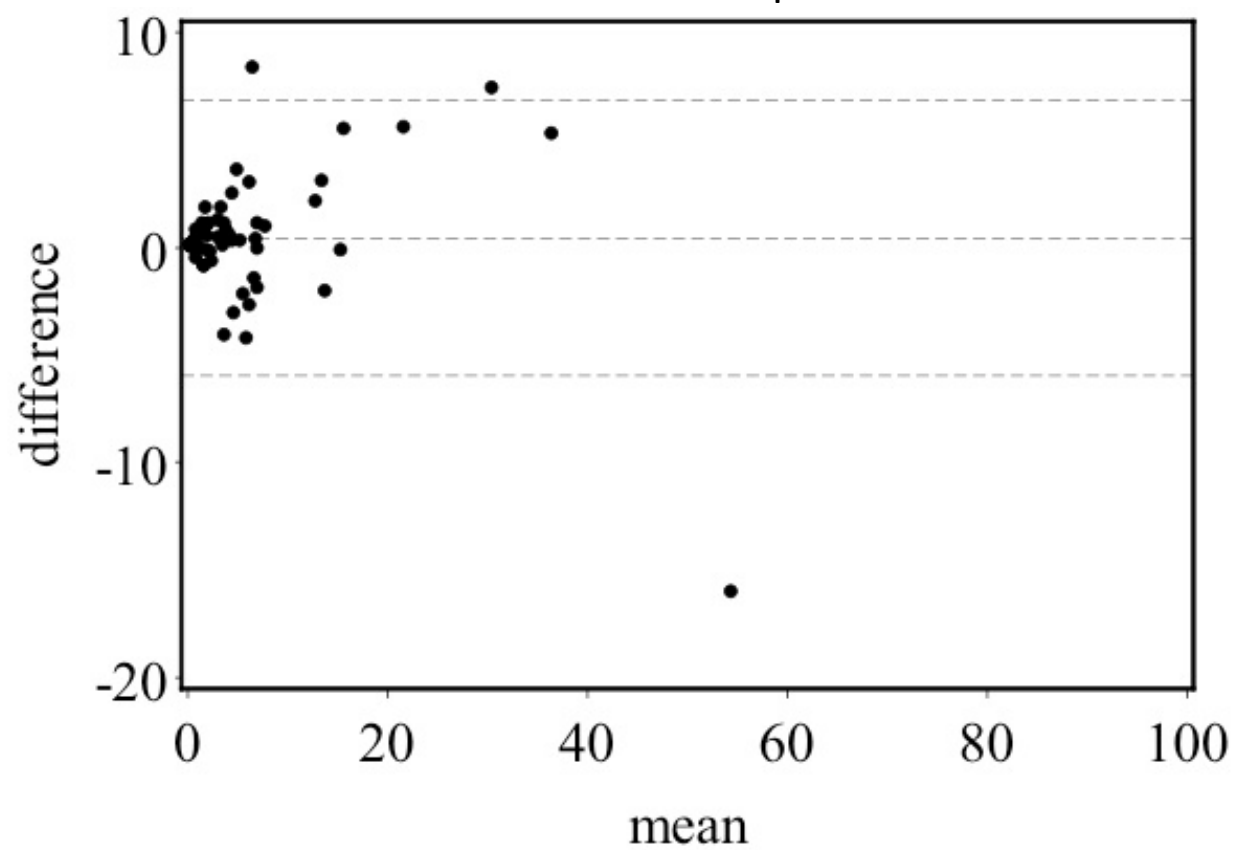

Supplement: Supplementary file 5 — Supplementary material 5 (PDF 94 kb) [file 10549_2017_4329_MOESM5_ESM.pdf]

A

Scatter plot

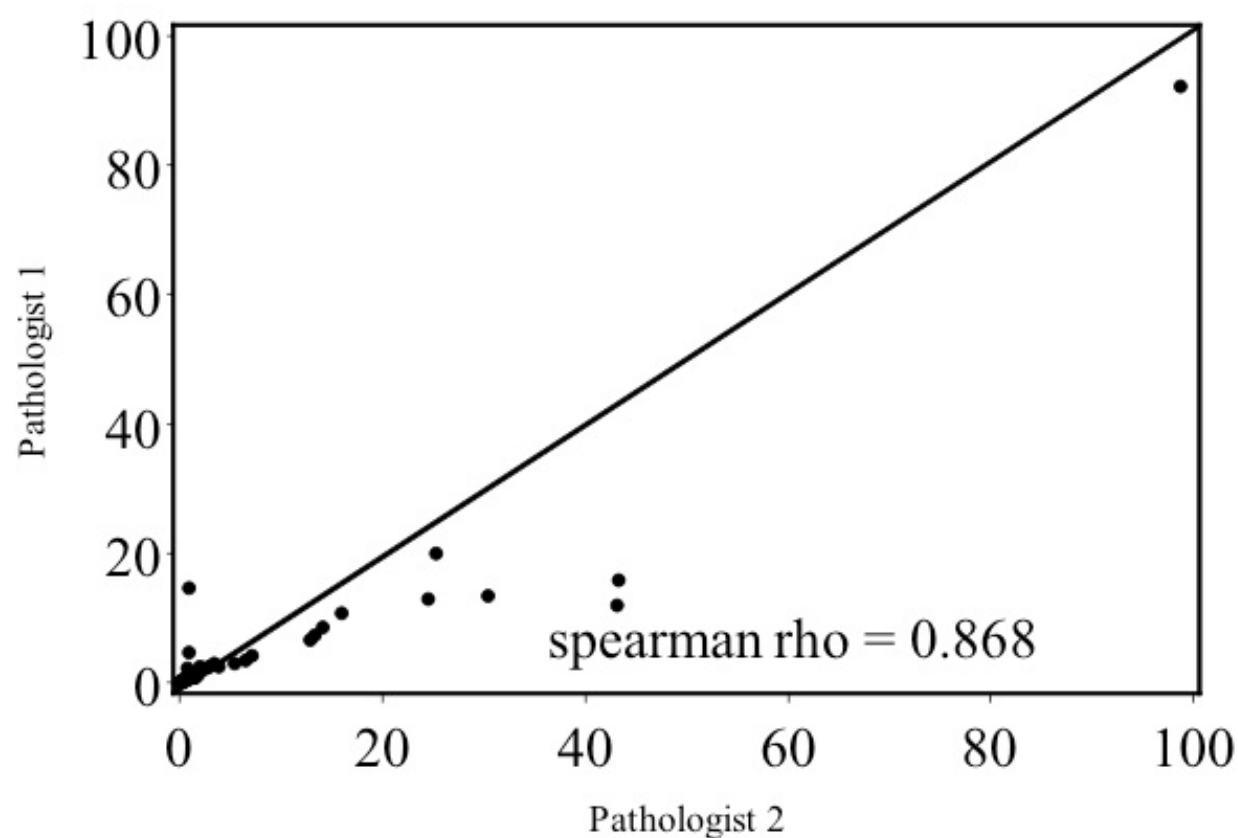

B

Bland Altman plot

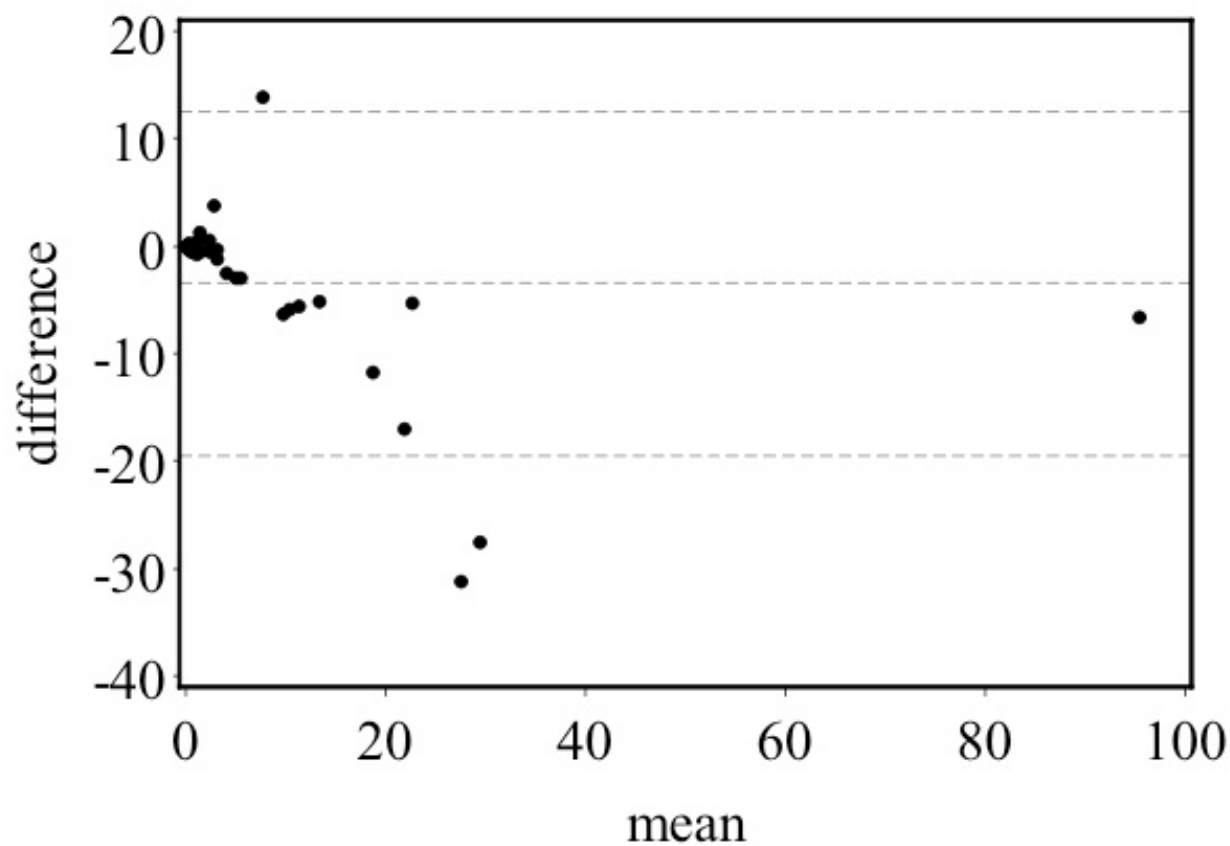

Supplement: Supplementary file 6 — Supplementary material 6 (PDF 93 kb) [file 10549_2017_4329_MOESM6_ESM.pdf]

A

Scatter plot

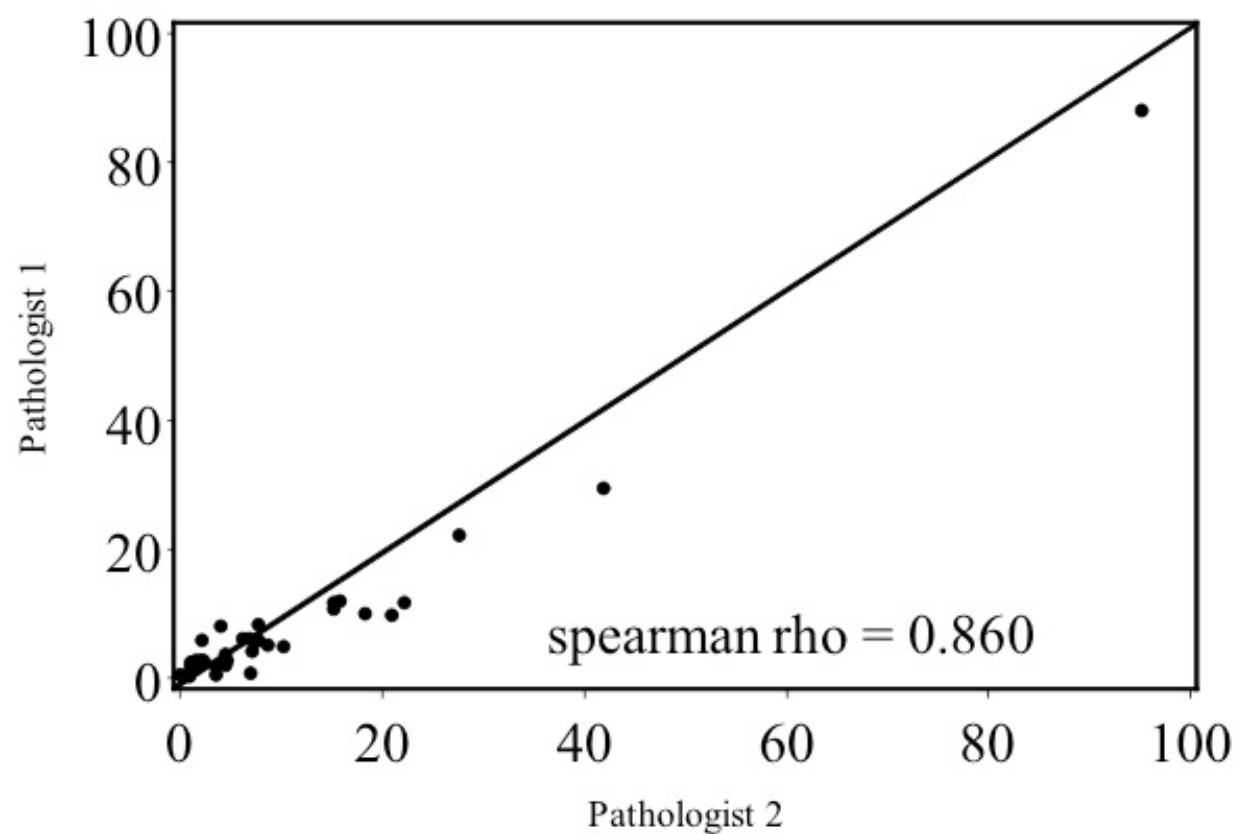

B

Bland Altman plot

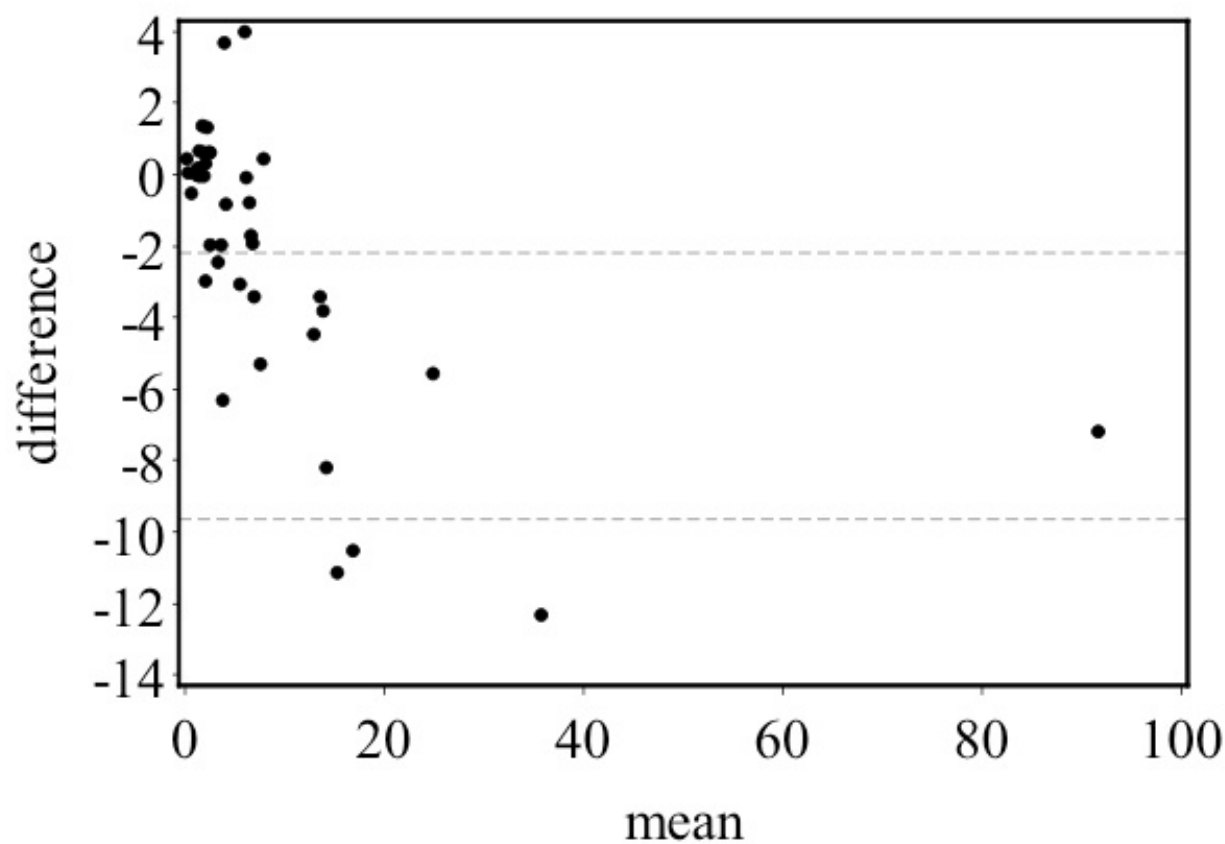

Supplement: Supplementary file 7 — Supplementary material 7 (PDF 95 kb) [file 10549_2017_4329_MOESM7_ESM.pdf]
